# Cobalt(II) chloride catalyzed one-pot synthesis of α-aminonitriles

**DOI:** 10.1186/1860-5397-1-8

**Published:** 2005-10-07

**Authors:** Surya K De

**Affiliations:** 1 Department of Medicinal Chemistry and Molecular Pharmacology, School of Pharmacy and Purdue Cancer Center, Purdue University, West Lafayette, IN 47907, USA

## Abstract

A simple and efficient method has been developed for the synthesis of α-aminonitriles by a one-pot three- component condensation of aldehydes, amines, and potassium cyanide in acetonitrile in the presence of a catalytic amount of CoCl_2_ at room temperature.

## Introduction

The addition of cyanide to imines (the Strecker reaction) [1] provides one of the most direct and viable routes for the synthesis of α-aminonitriles, which are useful intermediates for the synthesis of amino acids and nitrogen-containing heterocycles such as thiadiazoles, imidazoles, etc [2–3]. They are usually prepared by the nucleophilic addition of cyanide anion to imines. Numerous methods describing the preparation of α-aminonitriles have been reported in the literature employing acid catalysts such as InCl_3_, [4] BiCl_3_, [5] KSF clay, [6] Sc(OTf)_3_, [7] Cd(II)-salt, [8] Pt-salt, [9] and other. [10] However, there are still some drawbacks in these catalytic systems including low yields of products, long reaction times, harsh reaction conditions, the requirement for an inert atmosphere, the use of stoichiometric and/or relatively expensive reagents, [4–5,7–9] and also require tedious work-up leading to the generation of a large amount of toxic waste. Therefore, there is a need an efficient and inexpensive catalyst for the synthesis of α-aminonitriles.

In continuation of our work to develop new organic transformations, [11–17] I report herein that cobalt(II) chloride which acts as a mild Lewis acid might be a useful and inexpensive catalyst for the synthesis α-aminonitrile. Although cobalt(II) chloride has been extensively used as a mild catalyst for a plethora of organic transformations, [18–20] there are no examples of the use of cobalt(II) chloride as catalyst for the synthesis of α-aminonitriles.

## Results and discussion

**Scheme 1:**
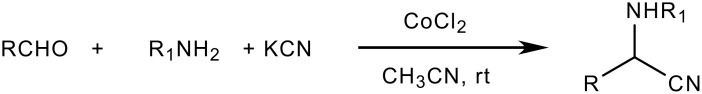


The treatment of benzaldehyde and aniline with KCN in the presence of a catalytic amount of CoCl_2_ afforded the corresponding 2-(*N*-anilino)-2-phenylacetonitrile in 91% yield. Similarly, a variety of aldehydes were coupled with a wide range of amines and potassium cyanide in a one-pot operation in the presence of a catalytic amount of CoCl_2_ at room temperature to give the corresponding α-aminonitriles in good to excellent yields (Scheme 1). Both aromatic and aliphatic aldehydes afforded excellent yields whereas ketones did not give any satisfactory yields. On the other hand, all types of primary and secondary amines are readily coupled in good yields. Moreover, acid sensitive aldehyde such as furfuraldehyde reacted in high yield. This method does not require any additives to promote the reaction. The results shown in Table 1 clearly indicate the scope and generality of the reaction with respect to various aldehydes and amines. One reaction was performed for the synthesis of 2-(*N*-Anilino)-2-phenylacetonitrile (entry 1) using trimethylsilyl cyanide instead of potassium cyanide as cyanide source to give the similar yield. It should be mentioned that both are toxic but potassium cyanide is cheaper than trimethylsilyl cyanide.

**Table 1:** Cobalt (II) chloride- catalyzed synthesis of α-amino nitriles with potassium cyanide

Entry	Aldehyde	Amine	Time (h)	Yield ^a^(%)

1	Benzaldehyde	Aniline	10	91
2	4-Chlorobenzaldehyde	Aniline	12	89
3	Decylaldehyde	Aniline	14	76
4	3-Methoxybenzaldehyde	Benzyl amine	10	85
5	Furfural	Aniline	12	80
6	Thiophene 2-carboxaldehyde	Benzyl amine	11	82
7	Benzaldehyde	Morpholine	12	74
8	Butyraldehyde	Pyrrolidine	10	80
9	2,4-Dimethoxybenzaldehyde	3,4,5-Trimethoxyaniline	11	81
10	4-Methoxybenzaldehyde	Aniline	12	83

^a^ Yields refer to pure isolated products and were characterized by spectral data.

## Conclusion

I have demonstrated a very simple, efficient, and practical method for the synthesis of α-aminonitriles through a one-pot three component coupling of aldehydes, amines, and potassium cyanide using a catalytic amount of cobalt(II) chloride. The major feature of this method is that it is truly a one-pot protocol that does not need a separate step to synthesize an imine for subsequent use. The advantages of this method include (a) operational simplicity, (b) no need any other additive to promote the reaction, (c) short reaction times, (d) the use of relatively cheap commercially available reagents, and (e) high yields of products.

## Supporting Information

File 1 Experimental details.
