# Supplementary material for: Cobalt(II) chloride catalyzed one-pot synthesis of α-aminonitriles
Source: Beilstein J Org Chem. 2005 Oct 7;1:8. doi: 10.1186/1860-5397-1-8 (PMC1399456; doi:10.1186/1860-5397-1-8)
Supplement: File 1 — Experimental details. [file Beilstein_J_Org_Chem-01-08-s001.doc]

**Experimental**

NMR spectra were recorded on a Bruker ARX 300 (300 MHz) instrument. Low resolution mass spectra (CI, EI) were recorded on a Finnigan 4000 mass spectrometer. High resolution mass spectra (HRMS, EI, CI, ESI) were recorded on Finnigan MAT XL95 mass spectrometer. Melting points were recorded on Buchi R-535 apparatus and are uncorrected. The reactions were monitored by TLC, and visualized with UV light followed by development using 15% phosphomolybdic acid in ethanol. All solvents and reagents were purchased from Aldrich with high -grade quality, and used without any purification. All yields refer to isolated products. All products are known ^3-7^ and were identified by comparison of their spectral data and physical properties with those of authentic samples.

**A typical procedure**

A mixture of benzaldehyde (212 mg, 2 mmol), aniline (186 mg, 2 mmol), and potassium cyanide (195 mg, 3 mmol) in acetonitrile (2 mL) was stirred at room temperature in the presence of CoCl_2_ (59 mg, 10 mol%). After completion of reaction (TLC), the reaction mixture was extracted with ethyl acetate (2x 20 mL). The organic layer was washed with water (20 mL), and brine (20 mL) respectively, then dried (MgSO_4_), and concentrated. The residue was chromatographed over silica gel (20% ethyl acetate in hexane) to afford the pure product. **Caution!** [*Potassium cyanide*](http://www.orgsyn.org/orgsyn/chemname.asp?nameID=32996) is highly toxic. Care should be taken to avoid direct contact of the chemical or its solutions with the skin, and impervious gloves should be worn to handle the reagent.

Product characterization data

2-(*N*-Anilino)-2-phenylacetonitrile (**Compound 1**, entry 1): Mp 83-85 ^O^C, lit.^3^ 84-85 ^O^C; ^1^H NMR (300 MHz, CDCl_3_) δ 4.02 (br s, 1 H), 5.41 (s, 1 H), 6.74 (d, *J* = 7.8 Hz, 2 H), 6.90 (t, *J* = 7.8 Hz, 1 H), 7.24 (t, *J* = 7.8 Hz, 2 H), 7.40-7.50 (m, 3 H); ^13^C NMR (75 MHz, CDCl_3_) δ 50.6, 114.8, 118.8, 120.6, 127.7, 129.7, 130.1, 130.3,134.5, 135.6; EIMS *m/z* 208 (M^+^), 180, 116, 91, 77, 55; HRMS calculated for C_14_H_12_N_2_ 208.1004, found 208.1006.

**Compound 2**: Mp 110-111 ^O^C, lit.^5^ 109-112 ^O^C; ^1^H NMR (300 MHz, CDCl_3_) δ 4.02 (s, 1 H), 5.39 (s, 1 H), 6.75 (d, *J* = 8 Hz, 2 H), 6.92 (t, *J* = 7.8 Hz, 1 H), 7.15 (t, *J* = 7.9 Hz, 2 H), 7.38 (d, *J* = 7.8 Hz, 2 H), 7.61 (d, *J* = 8 Hz, 2 H).

**Compound 3**: ^1^H NMR (300 MHz, CDCl_3_) δ 0.92 (t, *J* = 6.7 Hz, 3 H), 1.18-1.38 (m, 12 H), 1.50-1.64 (m, 2 H), 1.81-1.92 (m, 2 H), 3.82 (br s, NH), 4.02-4.16 (m, 1 H), 6.62 (d, *J* = 8 Hz, 2 H), 6.81 (t, *J* = 7.8 Hz, 1 H), 7.21 (t, *J* = 7.8 Hz, 2 H); EIMS *m/z* 258 (M^+^), 185, 155, 121, 77, 55; HRMS calculated for C_17_H_26_N_2_ 258.2095, found 258.2092.

**Compound 4**: ^1^H NMR (300 MHz, CDCl_3_) δ 1.84 (br s, 1 H), 3.76 (s, 3 H), 3.95 (AB, q, *J* = 13 Hz, 2 H), 4.64 (s, 1 H), 6.85 (dd, *J* = 2.4, 9 Hz, 1 H), 7.14-7.45 (m, 8 H); ^13^C NMR (75 MHz, CDCl_3_) δ 51.6, 53.9, 55.7, 113.4, 114.8, 119.2, 119.8, 128.5, 128.8, 129.7, 130.4, 136.7, 138.6, 160.4; EIMS *m/z* 252 (M+), 122, 91, 77; HRMS calculated for C_16_H_16_N_2_O 252.1262, found 252.1264.

**Compound 5**: Mp 68-69 ^O^C, lit.^3^ 68-70 ^O^C; ^1^H NMR (300 MHz, CDCl_3_) 4.06 (d, *J* = 8 Hz, 1 H), 5.40 (d, *J* = 8 Hz, 1 H), 6.40 (m, 1 H), 6.56 (m, 1 H), 6.80 (d, *J* = 8 Hz, 2 H), 6.91 (t, *J* = 8 Hz, 1 H), 7.26 (t, *J* = 8 Hz, 2 H), 7.42 (m, 1 H), EIMS *m/z* 198 (M^+^), 169, 155, 141, 115, 106, 92, 77, 51; HRMS calculated for C_12_H_10_N_2_O 198.0793, found 198.0791.

**Compound 6**: ^1^H NMR (300 MHz, CDCl_3_) δ 2.02 (br s, 1 H), 3.39 (AB, q, *J* = 13 Hz, 2 H), 4.91 (s, 1 H), 6.94-6.99 (m, 1 H), 7.21-7.46 (m, 7 H), EIMS *m/z* 228 (M^+^); HRMS calculated for C_13_H_12_N_2_S 228.0721, found 228.0725.

**Compound 7**: ^1^H NMR (300 MHz, CDCl_3_) δ 2.51-2.64 (m, 4 H), 4.68-4.79 (m, 5 H), 7.35-7.56 (m, 5 H); ^13^C NMR (75 MHz, CDCl_3_) δ 50.4, 62.8, 115.7, 128.5, 129.4, 129.8, 133.2; EIMS *m/z* 202 (M^+^); HRMS calculated for C_12_H_14_N_2_O 202.1106, found 202.1108.

**Compound 8**: ^1^H NMR (300 MHz, CDCl_3_) δ 0.98 (t, *J* = 7.7 Hz, 3 H), 1.42-1.52 (m, 2 H), 1.69-1.89 (m, 6 H), 2.62-2.71 (m, 4 H), 3.75 (t, *J* = 7.5 Hz, 1 H); ^13^C NMR (75 MHz, CDCl_3_) δ 11.6, 17.4, 21.4, 32.9, 47.9, 53.2, 94.4; EIMS *m/z* 152 (M^+^); HRMS calculated C_9_H_16_N_2_ 152.1313, found 152.1311.

**Compound 9**: ^1^H NMR (300 MHz, CDCl_3_) δ 3.76 (s, 3 H), 3.81 (s, 3 H), 3.82 (s, 6 H), 4.18 (br s, 1 H), 5.44 (s, 1 H), 6.01 (s, 2 H), 6.46-6.53 (m, 2 H), 7.39 (d, *J* = 9 Hz, 1 H); EIMS *m/z* 358 (M^+^); HRMS calculated for C_19_H_22_N_2_O_5_ 358.1529, found 358.1531.

**Compound 10**: ^1^H NMR (300 MHz, CDCl_3_) δ 2.41 (s, 3 H), 3.90 (s, 1 H), 5.41 (s, 1 H), 6.78 (d, *J* = 8 Hz, 2 H), 6.91 (t, *J* = 7.8 Hz, 1 H), 7.21-7.31 (m, 4 H), 7.50 (d, *J* = 8 Hz, 2 H); EIMS *m/z* 222 (M^+^), 176, 103, 77; HRMS calculated for C_15_H_14_N_2_ 222.1156, found 222.1158.
